# Cost-effectiveness of self-injected DMPA-SC compared with health-worker-injected DMPA-IM in Senegal^☆^^☆☆^

**DOI:** 10.1016/j.conx.2019.100012

**Published:** 2019-10-23

**Authors:** Mercy Mvundura, Laura Di Giorgio, Chloe Morozoff, Jane Cover, Marguerite Ndour, Jennifer Kidwell Drake

**Affiliations:** a PATH, PO Box 900922, Seattle, WA 98109, USA; b PATH, BP 15115, Dakar-Fann, Dakar, Senegal

**Keywords:** Cost-effectiveness, Economic evaluation, DMPA-SC, Injectable contraception, Self-injection, Family planning

## Abstract

**Objectives:**

To evaluate the cost-effectiveness of self-injected subcutaneous depot medroxyprogesterone acetate (DMPA-SC) compared to health-worker-administered intramuscular DMPA (DMPA-IM) in Senegal and to assess how including practice or demonstration injections in client self-injection training affects estimates.

**Study design:**

We developed a decision-tree model with a 12-month time horizon for a hypothetical cohort of 100,000 injectable contraceptive users in Senegal. We used the model to estimate incremental costs per disability-adjusted life year (DALY) averted. The analysis derived model inputs from DMPA-SC self-injection continuation and costing research studies and peer-reviewed literature. We evaluated the cost-effectiveness from societal and health system perspectives and conducted one-way and probabilistic sensitivity analyses to test the robustness of results.

**Results:**

Compared to health-worker-administered DMPA-IM, self-injected DMPA-SC could prevent 1402 additional unintended pregnancies and avert 204 maternal DALYs per year for this hypothetical cohort. From a societal perspective, self-injection costs less than health worker administration regardless of the training approach and is therefore dominant. From the health system perspective, self-injection is dominant compared to health worker administration if a one-page instruction sheet is used and one additional DMPA-SC unit is used for training and is cost-effective at $208 per DALY averted when two additional DMPA-SC units are used. Sensitivity analysis showed estimates were robust.

**Conclusions:**

Self-injected DMPA-SC averted more pregnancies and DALYs and cost less from the societal perspective compared to health-worker-administered DMPA-IM and hence is dominant. Using fewer DMPA-SC units for practice or demonstration improves cost-effectiveness of self-injection from the health system perspective.

**Implications:**

Evidence from Senegal shows that self-injection of DMPA-SC can be dominant or cost-effective from both health system and societal perspectives relative to DMPA-IM from health workers even if women practice injecting or health workers demonstrate with one or two DMPA-SC units. Evidence on whether practice or demonstration is required for client training would be useful.

## 1 Introduction

Francophone countries in West Africa have among the lowest rates of modern contraceptive use and highest rates of fertility and maternal mortality [1]. In recognition of the unique regional context, including struggling health infrastructure, the Ouagadougou Partnership (OP) was established in 2011 by the governments of nine Francophone West African countries, including Senegal, and their technical partners. The OP aims for better coordination between donors so as to optimize their family planning funding support to countries, and also for collaboration and cooperation at national and regional levels to address high rates of unmet family planning needs in the OP countries [2]. In Senegal, 18.9% of women use a modern method of family planning [3]; of these women, more than a third have chosen an injectable contraceptive, most commonly the intramuscularly administered depot medroxyprogesterone acetate (DMPA-IM). A new injectable contraceptive, subcutaneous DMPA (DMPA-SC), was introduced in Senegal in 2014. This easy-to-use product combines a single dose of the drug in a small reservoir with a needle in the Uniject™ injection system,^1^ enabling convenient administration by self-injection or by health workers with minimal training. Self-injection may be one contraceptive administration strategy that can increase access to contraceptives and reduce the unmet family planning needs, as targeted by the OP goals.

Self-injected DMPA-SC is a feasible and acceptable contraceptive option, as evidenced by a series of research studies [[4], [5], [6], [7], [8], [9]]. A Senegal-specific study found that 87% of the study population self-injected competently — without further health worker guidance — 3 months after being trained; the implications of these findings, both for women who self-injected successfully and for those who did not, are discussed elsewhere [4]. This study also found that 93% of women who tried self-injection expressed a desire to continue the practice, consistent with high acceptability reported elsewhere [4,5,10,11]. Four additional studies have demonstrated the benefits of this new delivery modality: self-injection improves 12-month continuation rates compared with health-worker-administered injections [[12], [13], [14], [15]].

A previous study evaluated the costs of administering injectable contraception using different delivery strategies in Burkina Faso, Uganda and Senegal [16]. Using data from Uganda and Senegal, the study found that self-injection reduces injectable contraception delivery costs for healthcare programs and women compared to health worker administration (by US $2.29 per client per year for Uganda and $1.08 for Senegal). However, self-injection delivery costs per client per year were higher in Senegal ($9.46) than Uganda ($7.83). The same study also evaluated costs of health worker administration of DMPA and found that costs were higher in Burkina Faso and Senegal compared to Uganda.

Another study evaluated the cost-effectiveness of self-injection using data from Uganda and found that self-injection can be cost-effective from a health system as well as a societal perspective because of the lower costs and the higher continuation rates compared with health worker administration [17]. The study found that if costs for self-injection increased, however, the delivery strategy would not be cost-effective from a health system perspective. A key health system cost driver was the self-injection training cost: using a client training aid formatted to be shorter and reduce printing costs, but still containing the same information as the longer one used in a research study, made self-injection cost-effective. As countries advance self-injection from the research setting into family planning programs, they also need information regarding whether client practice or health worker demonstration injections are necessary, given additional supply needs along with feasibility and cost considerations. It is also important to note that self-injection was cost-effective relative to health worker administration in Uganda from the societal perspective regardless of the training approach used, given the sizeable reductions in time and travel costs for women. Framing societal cost savings in terms of women's preferences, over 90% of women in Senegal identified saving time, and nearly half identified saving money as key motivations for opting to self-inject [4].

Given the unique regional context for OP countries and the higher costs of delivering injectable contraception that have been reported in Burkina Faso and Senegal [16], a cost-effectiveness analysis of self-injection in Senegal can yield important insights for decision-makers considering self-injection in those settings. Our objectives were to compare the cost-effectiveness of self-injected DMPA-SC with that of facility-based health-worker-administered DMPA-IM in Senegal and to provide evidence on self-injection training costs, including how the number of practice or demonstration units for training affects cost-effectiveness estimates. We aimed to provide evidence on whether the benefits of self-injection — measured through longer continuation rates and thus fewer unintended pregnancies — outweigh any additional costs incurred compared with health worker administration.

## 2 Methods

### 2.1 DMPA delivery strategies evaluated

We conducted this study in concert with the Senegal injectable contraceptive continuation study, where women who visited a clinic to receive an injectable contraceptive could choose to be trained to self-inject DMPA-SC or receive a DMPA-IM injection from a health worker [15]. (Health worker administration was the standard of care for injectable contraception in Senegal at the time, but acknowledging that it was not a direct comparison of the same method). Women who chose DMPA-IM had the injection administered by the health worker, who reminded them at the end of that visit to return in 3 months for their next injection. No additional follow-up or reminders were sent. Women who chose self-injection were trained by the health worker and practiced the injection technique on a salt-filled condom model using as many water-filled Uniject devices as needed. On average, women used four practice devices. Women deemed competent (performing five critical injection steps correctly) were supervised by the health worker as they self-injected their first dose and then were given three DMPA-SC units for independent self-injection, a calendar for scheduling subsequent injections and an instruction sheet. None of the study participants received additional reinjection reminders. We calculated costs based on the research intervention described above. However, practice injections using water-filled devices are no longer recommended, so practice or health worker demonstration now requires DMPA-SC units ($0.85 per unit compared with water-filled devices at $0.30 per unit) [18]. Therefore, we also evaluate how including additional DMPA-SC units for client training impacts the cost-effectiveness estimates.

### 2.2 The cost-effectiveness model

**Fig. 1:**
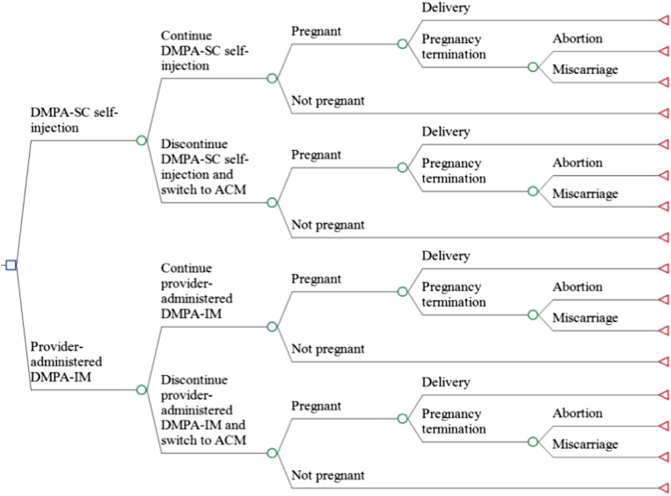
Decision-tree model to compare the costs and effectiveness of self-injected DMPA-SC versus health-worker-administered DMPA-IM.

To facilitate cross-country comparisons, we replicated methods used in our Uganda study [17]. We developed a static decision-tree model for a hypothetical cohort of 100,000 women in Senegal who self-injected DMPA-SC or received DMPA-IM from a health worker (Fig. 1). The cohort size was based on the estimated number of women of reproductive age in Senegal who used injectable contraceptives in 2017. We used a 1-year time horizon, as this was used in the Senegal continuation study [15]. We assumed that, at the beginning of this 12-month period, women were using these contraceptives due to a desire to prevent pregnancy.

The model assumed that when a woman was due for reinjection, she could choose either to continue with her current method or to discontinue and switch to another contraceptive method or to no method. We assumed that if the woman discontinued self-injection, she would not reinitiate self-injection at a later time within the 12 months. We also assumed that if the woman discontinued health-worker-administered DMPA-IM, she could not switch to self-injection (which was not routinely available in Senegal at the time). These assumptions were consistent with the continuation study design. Since our time horizon was a 1-year period and we were using a static model, we assumed that discontinuation happened at 6 months (midpoint). For discontinuers, we estimated the weighted costs and method effectiveness for the average contraceptive method (ACM) for the remaining 6-month period after discontinuation [19]. This ACM was calculated using unpublished data from the Senegal continuation study on the types of methods women switched to and the proportions of women switching to these, by injectable group [20]. Some of the women in the Senegal continuation study who discontinued self-injection chose DMPA-SC or DMPA-IM injections from a health worker, and we took this scenario into account when modeling the ACM effectiveness and costs estimates. All women in the model, whether they were using their originally elected injectable contraceptive, had switched methods or had discontinued contraception, had a probability of either becoming pregnant or not. Each pregnancy had a probability of ending with termination (miscarriage or abortion) or with delivery of an infant.

### 2.3 Perspectives and key model inputs

The cost-effectiveness analysis considered both health system and societal perspectives. For the health system perspective, costs for health-worker-administered DMPA-IM included commodity costs (injectable contraceptive, syringes and safety box), time cost for health workers to administer the contraceptive and treat side effects (if applicable), and drugs used for treatment of side effects (such as ibuprofen and oral contraceptives). For self-injection, commodity costs and drug costs were included, as were the time costs for health workers to train women to self-inject and treat side effects and the cost of self-injection training supplies. We assumed that women who continued for the year would use four units of DMPA. Key cost estimates used in this analysis were informed by a costing study we conducted in Senegal [16] (Table 1).

**Table 1:** Key cost inputs used in the Senegal DMPA cost-effectiveness model; costs are listed per client (in 2016 US $)

Parameter	Base case	Data source	Minimum and maximum values for the one-way sensitivity analysis^a^
**Contraceptive service delivery costs from the health system perspective**			
Direct medical costs for first visit for DMPA-SC self-injection at the health facility^b^ when four water-filled Uniject devices used per woman to practice/demonstrate the injection technique	$4.78	[16]	$2.39; $9.56^c^
Direct medical costs for first visit for DMPA-SC self-injection at the health facility when one DMPA-SC unit used per woman to practice/demonstrate the injection technique	$4.27	Calculated	$2.39; $9.56^c^
Direct medical costs for first visit for DMPA-SC self-injection at the health facility when two DMPA-SC units used per woman to practice/demonstrate the injection technique	$5.18	Calculated	$2.39; $9.56^c^
Direct medical costs for first visit for DMPA-SC self-injection at the health facility when three DMPA-SC units used per woman to practice/demonstrate the injection technique	$6.08	Calculated	$2.39; $9.56^c^
Direct medical costs for first visit for DMPA-SC self-injection at the health facility when four DMPA-SC units used per woman to practice/demonstrate the injection technique	$6.99	Calculated	$2.39; $9.56^c^
Direct medical costs for each subsequent DMPA-SC self-injection away from the facility	$0.88	[16]	$0.85^d^; $1.75
Direct medical costs of health-worker-administered DMPA-IM for four injections	$6.44	[16]	-
Direct medical costs for first DMPA-IM injection by a facility-based health worker^b^	$2.67	[16]	$1.34; $5.34
Direct medical costs for each subsequent DMPA-IM injection by a facility-based health worker	$1.26	[16]	$0.90^d^; $2.52
Direct medical costs of the ACM for 0.5 year after discontinuing DMPA-SC	$1.41	[35]	$0.71; $2.82
Direct medical costs of the ACM for 0.5 year after discontinuing DMPA-IM	$1.62	[35]	$0.81; $3.25
**Contraceptive service delivery costs from the societal perspective**			
Direct medical and direct nonmedical costs for first visit for DMPA-SC self-injection at the health facility^b^ when four water-filled Uniject devices used per woman to practice/demonstrate the injection technique	$5.67	[16]	$2.84; $15.14^c^
Direct medical and direct nonmedical costs for first visit for DMPA-SC self-injection at the health facility when one DMPA-SC unit used per woman to practice/demonstrate the injection technique	$5.15	[16]	$2.84; $15.14^c^
Direct medical and direct nonmedical costs for first visit for DMPA-SC self-injection at the health facility when two DMPA-SC units used per woman to practice/demonstrate the injection technique	$6.06	[16]	$2.84; $15.14^c^
Direct medical and direct nonmedical costs for first visit for DMPA-SC self-injection at the health facility when three DMPA-SC units used per woman to practice/demonstrate the injection technique	$6.97	[16]	$2.84; $15.14^c^
Direct medical and direct nonmedical costs for first visit for DMPA-SC self-injection at the health facility when four DMPA-SC units used per woman to practice/demonstrate the injection technique	$7.87	[16]	$2.84; $15.14^c^
Direct medical and direct nonmedical costs for each subsequent DMPA-SC self-injection away from the facility	$0.91	[16]	$0.85^d^; $1.82
Direct medical and direct nonmedical costs of health-worker-administered DMPA-IM for four injections	$9.46	[16]	-
Direct medical and direct nonmedical costs for first DMPA-IM injection by a facility-based health worker	$3.42	[16]	$1.73; $6.84
Direct medical and direct nonmedical costs for each subsequent DMPA-IM injection by a facility-based health worker	$2.02	[16]	$1.01; $4.04
Direct medical and direct nonmedical costs of the ACM for 0.5 year after discontinuing DMPA-SC	$1.78	[35]	$0.89; $3.56
Direct medical and direct nonmedical costs of the ACM for 0.5 year after discontinuing DMPA-IM	$2.49	[35]	$1.25; $4.98
**Direct medical costs of pregnancy outcomes**			
Birth and newborn care costs^e^	$100.81	Calculated using costs from the Impact 2 model [21]	$50.40; $201.62
Miscarriage	$20.02	Calculated using costs from the Impact 2 model [21]	$10.01; $40.04
Abortion	$13.86	Calculated using costs from the Impact 2 model [21]	$6.93; $27.71
Societal costs per woman after a pregnancy resulting in a live birth^f^	$219.13	Calculated by adding value of lost time to the costs for birth and newborn care above	$109.57; $438.26

a lognormal distribution was used in the probabilistic sensitivity analysis for all these cost inputs.

b This cost includes commodity costs (injectable contraceptive costs, syringes and safety box costs), time cost for health workers at health facilities for administering injectable contraceptives, and drugs used for treatment of side effects. For self-injection, it includes commodity costs, time costs for health workers to train women to self-inject and supervise the first injection, and self-injection training supplies costs.

c One single cost is used in the analysis at a time depending on the scenario. The same min / max values are used across the scenarios as a result.

d For the lower end of costs for the subsequent doses, these were truncated to be not lower than the commodity costs for the injectables.

e Includes delivery, antenatal care, postnatal care and newborn care costs.

f Includes delivery, antenatal care, postnatal care, and newborn care costs and productivity loss estimates assuming maternity leave of 14 weeks.

From a societal perspective, costs included the health system costs, women's travel costs, and the value of time spent reaching the clinic and waiting to be seen by a health worker. For DMPA discontinuation, which we assumed occurred at 6 months among discontinuers, we calculated costs for the remaining 6 months for the ACM, including commodity costs, health worker time for administering the contraceptives to which women switched and drugs for treating typical side effects. Women's time and travel costs were also accounted for in the ACM costs calculations from the societal perspective.

The analysis accounts for the probability of becoming pregnant and for the associated health system and societal costs, including prenatal care and delivery, miscarriage, pregnancy termination [21] and productivity costs to the woman having a live birth, assuming 14 weeks of maternity leave [22] (Table 1).

For continuation rates, we used data from the Senegal continuation study, where the 12-month continuation rate among women who self-injected DMPA-SC was 0.802 compared with 0.704 for women who received health-worker-administered DMPA-IM. Typical-use effectiveness data were obtained from the literature (Table 2) [23].

**Table 2:** Key inputs to estimate effectiveness, including contraceptive continuation rates and typical-use effectiveness

Indicator	Base case (rate)^a^	Data source	Minimum and maximum values for the one-way sensitivity analysis^a^
**Continuation rates**			
12-month continuation rate with DMPA-SC self-injection	0.802	[15]	0.70; 0.90
12-month continuation rate with DMPA-IM	0.704	[15]	0.60; 0.80
**Types of contraceptives to which women switched after discontinuing self-injection of DMPA-SC (among those who had already switched to another contraceptive or planned to do so within 30 days)**			
Oral contraceptive	8.3	[15]	See footnote^b^
Intrauterine device	0	[15]	
DMPA-IM or DMPA-SC administered by a health worker	56.7	[15]	
Implant	0.8	[15]	
Male condom	0	[15]	
Traditional method	0	[15]	
No method	34.2	[15]	
**Types of contraceptives to which women switched after discontinuing** health-worker-**administered DMPA-IM (among those who had already switched to another contraceptive or decided to switch)**			
Oral contraceptive	10.2	[15]	See footnote^b^
Intrauterine device	1.1	[15]	
DMPA-IM or DMPA-SC administered by a health worker	45.6	[15]	
Implant	1.6	[15]	
Male condom	0.5	[15]	
Traditional method	0.5	[15]	
No method	40.1	[15]	
**Contraceptive effectiveness rates [1** − **failure rate] of injectables and other contraceptives to which women switched after discontinuation (for 12 months of use in Senegal)**			
Injectable effectiveness	.986	[23]	.95; 1.00
Oral contraceptive	.923	[23]	See footnote^b^
Intrauterine device	.991	[23]	
Implant	.991	[23]	
Male condom	.962	[23]	
Traditional method (average of withdrawal and periodic abstinence)	.919	[23]	
No method	.69	[21]	
**Weighted average effectiveness of the ACM to which women switched**			
ACM effectiveness (typical use) among women who discontinued self-injection of DMPA-SC	.880	Calculated	.82; .95
ACM effectiveness (typical use) among women who discontinued health-worker-administered DMPA-IM	.867	Calculated	.80; .91
**Probability of pregnancy outcomes**			See footnote^c^
Probability of a delivery	.76	[27]	.62; .82^c^
Probability of a miscarriage	.16	[27]	See footnote^c^
Probability of an abortion	.8	[27]	See footnote^c^

a Beta distributions were assumed for the sensitivity analysis, with parameter values of *α* = 2 and *β* = 2.

b These percentages are correlated and impact the average contraceptive method effectiveness. We evaluated the impact of these variables by varying it such that women would switch to either less effective or more effective methods after discontinuing self-injection of DMPA-SC or health worker administration of DMPA-IM, as relevant.

c These probabilities are interrelated and sum to 100%. The one-way sensitivity analysis was done by varying the percentage of pregnancies resulting in a delivery while holding constant the percentage resulting in an abortion and adjusting the percentage resulting in a miscarriage so that these three percentages add up to 100%.

### 2.4 Analysis

For each hypothetical cohort of women self-injecting DMPA-SC or receiving DMPA-IM from a health worker, we estimated the health system and societal costs of receiving the contraceptive services and the costs associated with any unintended pregnancies and their outcomes. We calculated the number of pregnancies and the maternal disability-adjusted life years (DALYs) averted in both groups. One DALY can be thought of as 1 lost year of “healthy” life, and DALYs are the sum of years of life lost due to premature mortality and the years lost due to disability for people living with the health condition or its consequences [24]. For pregnancy, the majority of DALYs lost are due to premature mortality attributable to pregnancy-related conditions (or maternal disorders). These maternal disorders include hemorrhage, sepsis and other maternal infections, abortions and miscarriages, hypertensive disorders, ectopic pregnancy, and obstructed labor and uterine rupture. Using data estimating that in 2016 an average of 105,027 DALYs were lost in Senegal due to pregnancy-related health conditions [25], we calculated the maternal DALYs lost per pregnancy (0.15) by dividing the estimated DALYs lost due to maternal disorders by the estimated annual number of pregnancies in Senegal, based on the number of live births, adjusted for pregnancy termination [26,27].

The main study outcomes were the incremental costs per maternal DALY averted over a 1-year time horizon, which were calculated as the difference in costs divided by the difference in effectiveness (DALYs averted) and evaluated against a cost-effectiveness threshold for Senegal of $544 per DALY averted [28]. This conservative threshold is lower than a traditional threshold based on the gross domestic product (GDP) per capita (US$958 in 2016 for Senegal) [29]. We conducted the analysis using Excel 2016 (Microsoft, Redmond, WA, USA). All costs are presented in 2018 US $.

### 2.5 Sensitivity analysis

We conducted one-way and probabilistic sensitivity analyses on all key inputs to explore the robustness of the results, given the uncertainty of inputs. One-way sensitivity analysis evaluates how the cost per DALY averted changes when we change one model input at a time. We conducted this analysis in Excel using the minimum and maximum values shown in Table 1, Table 2, allowing costs to vary 50% to 200% from the base case value. We conducted the probabilistic sensitivity analysis using @Risk software (Palisades Corporation, Ithaca, NY, USA). Probabilistic sensitivity analyses are multiway sensitivity analyses using simulation methods. We assigned probability distributions to all key model inputs and evaluated cost inputs assuming a lognormal distribution to account for skewness of costs, assuming probabilities to follow a beta distribution, as in previous studies [17,[30], [31], [32]]. We drew the set of key input values by randomly sampling from each distribution and ran the model 50,000 times to evaluate the robustness of the estimates.

### 2.6 Ethical approval

This study used data from a costing study [16] and the Senegal continuation study [15], both of which had ethical approval from the Research Determination / Ethics Committee at PATH and from the Comité National d'Ethique pour la Recherche en Santé in Senegal. This study was approved as part of the above referenced ethical approvals.

## 3 Results

### 3.1 Base case analyses

Due to the higher contraceptive continuation rate among women who self-injected DMPA-SC versus health-worker-administered DMPA-IM, our model estimated that for a hypothetical cohort of 100,000 women, self-injection could avert an additional 1402 pregnancies and 204 maternal DALYs over a 1-year period (Table 3). We used the IMPACT 2 model [21] to validate our model results for women receiving provider administered DMPA-IM and our estimates align. When taking a societal perspective (i.e., including health system costs and women's travel and time costs), self-injection of DMPA-SC would cost less than health-worker-administered DMPA-IM in all scenarios evaluated. Self-injection is therefore a dominant strategy (i.e., a strategy that costs less and has better health impact) from a societal perspective compared with health-worker-administration (Table 3).

**Table 3:** Effectiveness, costs and incremental cost-effectiveness estimates for a hypothetical cohort of approximately 100,000 injectable users in Senegal for a 1-year time horizon (in 2016 US$)

**Indicator**	Pregnancies averted	Maternal DALYs averted	Costs for health-worker-administered DMPA-IM	Costs for DMPA-SC when four water-filled Uniject devices are used for practice/demonstration	Costs for DMPA-SC when one additional DMPA-SC unit is used for practice/demonstration	Costs for DMPA-SC when two additional DMPA-SC units are used for practice/demonstration	Costs for DMPA-SC when three additional DMPA-SC units are used for practice/demonstration	Costs for DMPA-SC when four additional DMPA-SC units are used for practice/demonstration
**Societal: programmatic implementation**^a^								
DMPA-SC	26,942	3917		$1,401,652	$1,351,082	$1,440,254	$1,529,425	$1,617,617
DMPA-IM	25,540	3713	$1,696,757					
Difference compared to DMPA-IM	1402	204		($295,105)^b^	($345,675)	($256,503)	($167,332)	($79,140)
Incremental cost-effectiveness ratio per DALY averted^c^				Self-injected DMPA-SC is dominant^b^	Self-injected DMPA-SC is dominant	Self-injected DMPA-SC is dominant	Self-injected DMPA-SC is dominant	Self-injected DMPA-SC is dominant

**Health system: programmatic implementation**^a^								
DMPA-SC	26,942	3917		$992,356	$942,381	$1,031,553	$1,119,745	$1,208,916
DMPA-IM	25,540	3713	$988,757					
Difference compared to DMPA-IM	1,402	204		$3,744	($46,376)	$42,796	$130,988	$220,159
Incremental cost-effectiveness ratio per DALY averted^c^				$18/DALY averted	Self-injected DMPA-SC is dominant	$208	$644	$1080

a Programmatic design: the lower-cost training aid is used as the self-injection training aid.

b Dollar amounts in parenthesis reflect incremental cost savings that occur when the costs of self-injected DMPA-SC are lower than those for health-worker-administered DMPA-IM.

c We do not report negative incremental cost-effectiveness ratios, which occur when self-injection of DMPA-SC costs less and averts more DALYs than health worker administration of DMPA-IM.

Under a health system perspective, the 12-month total costs for contraceptive service delivery and pregnancy-related outcomes would be approximately $993,000 for self-injection of DMPA-SC, when the four water-filled devices are used for training (per the research intervention), and $989,000 with health worker administration of DMPA-IM (Table 3). The incremental cost-effectiveness ratios of self-injection of DMPA-SC versus health-worker-administration of DMPA-IM were estimated at $18 per DALY averted. Whether using the upper end of the conservative cost-effectiveness threshold for Senegal ($544) or the traditional threshold ($958), self-injection of DMPA-SC at $18 per DALY averted would be cost-effective compared to health worker administration of DMPA-IM.

In regard to practice or demonstration injections, Table 3 shows that from the health system perspective, if each self-injecting woman used one DMPA-SC unit for practice or observed a health worker demonstrate with one DMPA-SC unit, self-injection could cost less than health-worker-administered DMPA-IM and would be the dominant strategy. If each client training used two DMPA-SC units for practice or demonstration, self-injection would be cost-effective when applying the conservative threshold for cost-effectiveness ($544).

### 3.2 Sensitivity analysis

**Fig. 2:**
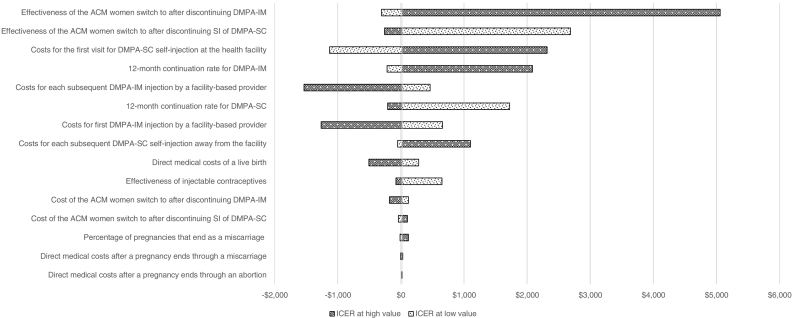
One-way sensitivity analysis for the cost-effectiveness of self-injection of DMPA-SC compared with health-worker-administered DMPA-IM from the health system perspective. A one-way sensitivity analysis evaluates the impact on the incremental cost-effectiveness ratio (ICER) of varying one model input while holding all other inputs constant. Key model inputs were varied, and the figure shows the 15 input values that had the most impact on the cost-effectiveness estimates. The wider the bars, the more the variation in the input values impacts the cost-effectiveness estimates. If using low/high values of the input increases the ICER, this means that low/high values of the input make self-injection less favorable. Similarly, if using low/high values of the input decreases the ICER, this means that low/high values of the input make self-injection more favorable.

Fig. 2 shows results for the one-way sensitivity analysis and identifies the most influential variables driving the cost-effectiveness estimates from the health system perspective. Among these variables, the two most influential were the effectiveness of the ACM women switched to after discontinuing their injectable (either DMPA-IM from the health worker or DMPA-SC self-injection). Varying this input from 0.80 to 0.91 for women who discontinued health worker administration of DMPA-IM resulted in incremental cost per DALY averted estimates ranging from −$308 to $5058. Hence, if women who discontinue DMPA-IM switch to methods that are less effective, this improves the cost-effectiveness of self-injection. Similarly, varying the effectiveness of the ACM women switched to after discontinuing self-injection from 0.82 to 0.95 resulted in incremental cost per DALY averted estimates ranging from $2687 to −$261, showing that if women who discontinue self-injection switch to less effective methods, this makes self-injection not cost effective. The third most influential variable was the cost of the first visit for women self-injecting DMPA-SC: the higher the cost for this visit, the less cost-effective the self-injection of DMPA-SC. The range used for this cost considered scenarios for varying the type and number of units used to practice or demonstrate injection technique.

**Fig. A1:**
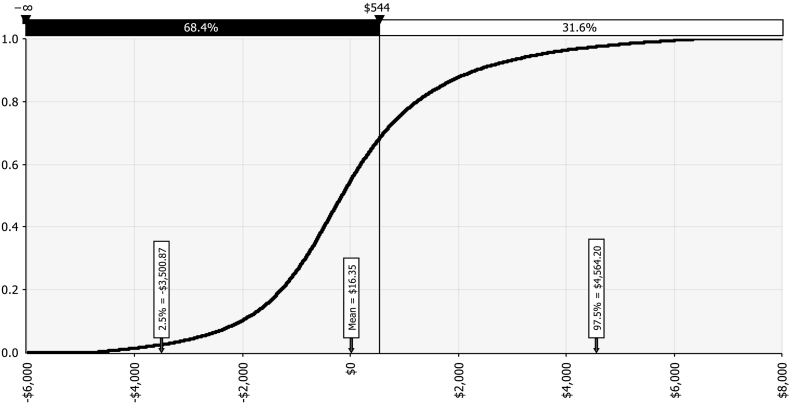
Cumulative ascending graph for the probabilistic sensitivity analysis on the cost-effectiveness of self-injection of DMPA-SC compared to health-worker-administered DMPA-IM from the health system perspective using the conservative cost-effectiveness threshold of $544 for Senegal [28].

The probabilistic sensitivity analysis showed that in 68.4% of iterations, self-injection of DMPA-SC was likely to be cost-effective from the health system perspective compared with health worker administration of DMPA-IM when using $544 as the threshold for cost-effectiveness (Appendix Fig. A1). The mean and 95% confidence intervals for this analysis were $16.35 [−$3501 to $4564]. When the traditional threshold was used, interventions that were less than three times the per capita GDP ($2874) were considered cost-effective, and self-injection of DMPA-SC was cost-effective in 93% of the iterations from the health system perspective (results not shown). For the societal perspective, the probabilistic sensitivity analysis showed that, in 88.6% of iterations, self-injection of DMPA-SC was likely to be cost-effective when using the conservative threshold ($544) and it would be cost-effective in 94.4% of the iterations if the traditional threshold was used (results not shown).

## 4 Discussion

Self-injection of DMPA-SC is one strategy that can help countries in Francophone West Africa work toward the goal of increasing access of contraceptives for women and hence increase the number of modern contraceptive users [1] and injectable continuation rates. This study demonstrates that self-injection of DMPA-SC is cost-effective compared with health-worker-administered DMPA-IM even when training uses DMPA-SC units for practice. Our findings are similar to those from Uganda [17], strengthening the evidence that self-injection is cost-effective and suggesting that the finding may hold true across countries (including others in Francophone West Africa) with different populations of women using contraception, varied costs of health care services and other variations.

A recent analysis found that investing $16.94 per capita per year in the Ouagadougou Partnership countries — which include Senegal — would address unmet need for contraception and provide women and newborns with essential care [1]. For new contraceptive innovations like self-injection to be included in these types of estimates and investments, program costs must be affordable for national health budgets. Because the costs of self-injection training were one of the key drivers of the cost-effectiveness estimates, reductions in the costs of training can contribute to improved cost-effectiveness. As program designs for self-injection evolve in pursuit of scale and sustainability, costs and their links to outcomes must be a consideration, and our study provides some insight into how self-injection training may be adjusted to achieve cost effectiveness.

A recent editorial on self-injection noted that: “Self-administration of DMPA-SC is feasible, acceptable, effective, and improves continuation… [now] what is most needed is implementation research to analyze how self-administration is implemented in practice and to understand the barriers and facilitators to successful implementation [33].” This analysis contributes to an understanding of self-injection program implementation with important insights on the benefits of a lower-cost training aid and the cost differentials and tradeoffs to consider around including practice or demonstration injections in a program. An evaluation is under way in Uganda to assess whether women can competently learn to self-inject through different training formats, such as instructional videos, group training, training in the community or through private-sector sites, and health worker demonstration and/or coaching, in lieu of practice injections. In addition, contraceptive self-injection falls into the category of a self-care intervention or “activities undertaken by individuals, families, and communities with the intention of enhancing health, preventing disease, limiting illness, and restoring health… undertaken by lay people on their own behalf either separately or in collaboration with health professionals” [34]. As Senegal and other countries strive to attain universal health coverage, an economic lens on self-care will be critical. Self-injection reduces time and travel costs for women, making it appealing for clients. Yet health systems must provide support and enable success for women who are exercising self-care, in the context of constrained national health budgets.

As published elsewhere [15], the profiles of self-injectors in the continuation study differed at baseline from those who received injections from providers (although self-injection remained a significant driver of continuation even controlling for these differences). For example, self-injectors had higher socioeconomic status and more years of education. More research is needed to understand the reasons behind these differences and can help inform family planning program strategies that make innovative self-care approaches like self-injection equally appealing and accessible to women who face barriers due, for example, to limited literacy — potentially further improving the cost-effectiveness of self-injection.

This study has several limitations. First, we took a 1-year time horizon, so training costs were not spread over a longer period, which underestimates the cost-effectiveness. We also took a conservative approach to estimating effectiveness by including only the maternal DALYs averted and not neonatal DALYs, which would improve cost-effectiveness estimates. Because the cost data were collected in a research setting, the analysis does not include the cost of training health workers who train women to self-inject and other self-injection introduction costs. The study also may have underestimated the probability of alternative pregnancy outcomes due to reliance on secondary data, though the sensitivity analysis showed that these variables have little impact on the cost-effectiveness results. We also assumed that discontinuation of self-injected DMPA-SC or provider administered DMPA-IM occurred at 6 months as per the requirements of a static model. However, discontinuation may occur at different times and also may occur at different times for the two administration methods. The impact of this assumption should be evaluated in future studies. The model also assumed any women getting pregnant would only be pregnant once during the 12-month analysis period; while repeat pregnancies may be theoretically possible, the delayed return to fertility following DMPA use makes this assumption reasonable. This study did not explore the impact of varying the number of DMPA-SC units self-injecting women might take home. This should be done in further research to explore the impact on health workers' time and women's time/travel costs; women's continuation; and ultimately, the overall cost-effectiveness. Finally, it is possible that cost-effectiveness for the full population of women of reproductive age might vary from the population included in this study, which was primarily recruited through clinics. For example, it is possible that continuation differences between self-injection and health worker administration are less distinct for women who are served by more accessible community health workers or private-sector outlets such as pharmacies.

The following are the supplementary data related to this article.

## Footnotes

1 Uniject is a trademark of BD.
